# GMMA as an Alternative Carrier for a Glycoconjugate Vaccine against Group A *Streptococcus*

**DOI:** 10.3390/vaccines10071034

**Published:** 2022-06-28

**Authors:** Elena Palmieri, Zoltán Kis, James Ozanne, Roberta Di Benedetto, Beatrice Ricchetti, Luisa Massai, Martina Carducci, Davide Oldrini, Gianmarco Gasperini, Maria Grazia Aruta, Omar Rossi, Cleo Kontoravdi, Nilay Shah, Fatme Mawas, Francesca Micoli

**Affiliations:** 1 GSK Vaccines Institute for Global Health (GVGH), Via Fiorentina 1, 53100 Siena, Italy; elena.x.palmieri@gsk.com (E.P.); roberta.x.di-benedetto@gsk.com (R.D.B.); beatrice.x.ricchetti@gsk.com (B.R.); luisa.x.massai@gsk.com (L.M.); martina.x.carducci@gsk.com (M.C.); davide.x.oldrini@gsk.com (D.O.); gianmarco.x.gasperini@gsk.com (G.G.); maria-grazia.x.aruta@gsk.com (M.G.A.); omar.x.rossi@gsk.com (O.R.); 2 The Sargent Centre for Process Systems Engineering, Department of Chemical Engineering, Imperial College London, South Kensington Campus, London SW7 2AZ, UK; z.kis@sheffield.ac.uk (Z.K.); cleo.kontoravdi98@imperial.ac.uk (C.K.); n.shah@imperial.ac.uk (N.S.); 3 Department of Chemical and Biological Engineering, The University of Sheffield, Mappin Street, Sheffield S1 3JD, UK; 4 The National Institute for Biological Standards and Control (NIBSC), South Mimms EN6 3QG, UK; James.Ozanne@nibsc.org (J.O.); Fatme.Mawas@nibsc.org (F.M.)

**Keywords:** Group A *Streptococcus*, GMMA, glycoconjugate, Group A Carbohydrate

## Abstract

Group A *Streptococcus* (GAS) causes about 500,000 annual deaths globally, and no vaccines are currently available. The Group A Carbohydrate (GAC), conserved across all GAS serotypes, conjugated to an appropriate carrier protein, represents a promising vaccine candidate. Here, we explored the possibility to use Generalized Modules for Membrane Antigens (GMMA) as an alternative carrier system for GAC, exploiting their intrinsic adjuvant properties. Immunogenicity of GAC-GMMA conjugate was evaluated in different animal species in comparison to GAC-CRM_197_; and the two conjugates were also compared from a techno-economic point of view. GMMA proved to be a good alternative carrier for GAC, resulting in a higher immune response compared to CRM_197_ in different mice strains, as verified by ELISA and FACS analyses. Differently from CRM_197_, GMMA induced significant levels of anti-GAC IgG titers in mice also in the absence of Alhydrogel. In rabbits, a difference in the immune response could not be appreciated; however, antibodies from GAC-GMMA-immunized animals showed higher affinity toward purified GAC antigen compared to those elicited by GAC-CRM_197_. In addition, the GAC-GMMA production process proved to be more cost-effective, making this conjugate particularly attractive for low- and middle-income countries, where this pathogen has a huge burden.

## 1. Introduction

Group A *Streptococcus* (GAS) is a Gram-positive human pathogen causing hundreds of millions of infections worldwide every year, from the most common superficial infections (pharyngitis, impetigo) to more severe invasive diseases [1]. GAS can spread into deep tissues or the bloodstream, causing sepsis, necrotizing fasciitis or streptococcal toxic shock syndrome, all potentially life-threatening conditions [2]. Furthermore, autoimmune diseases, e.g., acute post-streptococcal glomerulonephritis, acute rheumatic fever and rheumatic heart disease (RHD), have been associated in repeated GAS infections. RHD mostly affects adolescents and young adults, in particular pregnant women, with an estimated global prevalence of 33 million cases in 2015 [3,4,5]. GAS is also an important driver of antibiotic use [6], potentially contributing to increased resistance of other bacteria-colonizing humans [7].

The development of a vaccine against GAS is rather challenging because GAS infections are guided by several virulence factors and toxins varying across different serotypes [8,9], and no licensed vaccine is currently available.

A conserved and highly abundant cell wall component (30–50% by weight, [10]) is Group A Carbohydrate (GAC), which is constituted by a polyrhamnose backbone with N-Acetylglucosamine (GlcNAc) alternating at the side chain [11], and it is essential for GAS survival [12]. It has been shown that human anti-GAC antibodies confer protection against GAS throat colonization and promote bacterial phagocytosis [13,14]. For these reasons, GAC has been identified as a potential antigen for a candidate vaccine against GAS.

Covalent linkage of polysaccharides (PS), typically T-cell independent antigens, to a protein carrier, a source of T-cell epitopes, enables antibody class-switching and generation of immunological memory [15], making the resulting glycoconjugates effective in infants. This strategy has been applied to GAC, which has been conjugated to classic carrier proteins such as tetanus toxoid (TT) [13] or CRM_197_, a non-toxic mutant of diphtheria toxin [16,17]. TT-GAC conjugate was able to confer protection to immunized mice in a challenge model using two different M serotype GAS strains [13].

TT and CRM_197_ are being used for a large number of glycoconjugate vaccines, raising concerns about possible immune interference. Numerous pre-clinical and clinical studies have highlighted the so-called carrier-induced immune suppression effect. Anti-carrier antibody responses may impact the boost effect of the same vaccine or the response to other vaccines containing the same carrier [18], making desirable the identification of alternative carrier proteins or carrier systems [19].

To this end, three conserved GAS proteins (Streptolysin O, SpyAD and SpyCEP), selected as potential vaccine candidates [20], have been tested as carriers for GAC in mice, inducing an anti-GAC IgG response comparable to that of the more traditional CRM_197_ conjugate [16]. However, conjugation of GAC negatively impacted the anti-protein responses. In addition, fragments of Group A streptococcal C5a peptidase, another highly conserved surface virulence factor, have been proposed as carrier proteins for a glycoconjugate vaccine against GAS [21].

Recently, nanoparticle systems combining high valency presentation of the antigens with the physico-chemical properties of nano-sized particles have been investigated for the display of polysaccharides [19]. Among these systems, Outer Membrane Vesicles (OMV) have been proposed, which also possess intrinsic adjuvant properties [22,23,24]. GMMA (Generalized Modules for Membrane Antigens) are OMV derived from bacteria genetically engineered to enhance their release and to reduce the risk of reactogenicity [25]. The GMMA manufacturing process is simple, robust and cost-effective, thus potentially leading to affordable vaccines, especially for low- and middle-income countries (LMIC) [26]. GMMA have been exploited for the presentation of polysaccharides, showing the ability to induce similar anti-polysaccharide-specific IgG response to more traditional glycoconjugates often with stronger functionality in animal models [27,28]. In this work, we explored the possibility of using GMMA as an alternative carrier system for GAC, comparing its immunogenicity to that of a more traditional glycoconjugate in different animal models. In addition, a techno-economic analysis comparing the conjugation processes with the two different carriers has been performed. As a traditional carrier, we selected CRM_197_, one of the most commonly used carrier proteins for commercial glycoconjugates [29], already successfully tested for GAC [16,17]. Differently from TT, also widely used as carrier protein and tested for GAC as well [13,30], CRM_197_ does not require detoxification with formaldehyde, and consistent and homogeneous preparations can be readily obtained.

## 2. Materials and Methods

### 2.1. Materials

The following chemicals were used in this work: sodium chloride (NaCl) (Merck, Darmstadt, Germany), sodium phosphate monobasic (NaH_2_PO_4_), sodium phosphate dibasic (Na_2_HPO_4_), boric acid solution, deoxycholate (DOC), hydrochloric acid (HCl), sodium periodate (NaIO_4_), sodium sulfite (Na_2_SO_3_), sodium borohydride (NaBH_4_), sodium cyanoborohydride (NaBH_3_CN), phosphate buffered saline tablets (PBS) (Honeywell Fluka, Charlotte, NC, USA), glacial acetic acid (Carlo Erba reagents, Milan, Italy), dithiothreitol (DTT) (Invitrogen, Waltham, MA, USA).

GAC was chemically extracted with nitrite/glacial acetic acid treatment from a M protein-mutant strain (GAS51∆M1) derived from the wild-type strain HRO-K-51 kindly provided by the University of Rostock [31]. Extracted GAC was purified by tangential flow filtration and anionic exchange chromatography, as previously described [17]. Purified GAC contained no hyaluronic acid, <4% protein and <1% DNA impurities (w/w with respect to GAC) and showed an average molecular size of 7.0 kDa, corresponding to an average of 14 repeating units per chain, as estimated by HPLC-SEC analysis (TSK gel G3000 PWXL column) using a calibration curve of dextrans (5, 25, 50, 80, 150 kDa, Merck, Darmstadt, Germany).

CRM_197_ recombinant protein was obtained from GSK R&D (Siena, Italy).

GMMA were produced from *Salmonella enterica* serovar Typhimurium isolate 1418 (LT-2 collection, University of Calgari) Δ*tolR::*FRT Δ*rfbU-P::aph*. Δ*tolR* and Δ*rfbU-P* mutations were introduced to increase vesicles release and to disrupt OAg biosynthesis, respectively, sequentially substituting the genes of interest with the kanamycin resistance cassette *aph* through homologous recombination making use of the lambda red recombineering system [32]. For what concerns *tolR* mutation, the antibiotic cassette was removed using the pCP20 plasmid, as previously described [32]. Bacterial strain was grown at 30 °C in liquid Luria–Bertani (LB) medium in a rotary shaker for 16 h. Overnight cultures were diluted in HTMC medium (15 g/L glycerol, 30 g/L yeast extract, 0.5 g/L MgSO_4_, 5 g/L KH_2_PO_4_, 20 g/L K_2_HPO_4_) to an optical density at 600 nm (OD600) of 0.3 and grown at 30 °C in a rotary shaker for 8 h using baffled flasks with a liquid-to-air volume ratio of 1:5. GMMA were then purified from culture supernatant and characterized as previously reported [33]. High-Performance Anion-Exchange Chromatography coupled to Pulsed Amperometric Detector (HPAEC-PAD) analysis was used to verify the absence of OAg on GMMA. GMMA were free of soluble proteins and residual DNA as checked by High Performance Liquid Chromatography—Size Exclusion (HPLC-SEC, fluorescence emission and A260 profiles respectively). Particle size was of 49 nm (Z average diameter by Dynamic Light Scattering, DLS) [33].

### 2.2. GAC Glycoconjugates Synthesis and Characterization

#### 2.2.1. GAC-CRM197 Conjugate

GAC-CRM_197_ conjugate was produced as follows. GAC (~2 mg/mL) was incubated with 8 mM NaIO_4_ in phosphate buffer at pH 7.2 at 25 °C for 30 min. NaIO_4_ excess was then quenched with 16 mM Na_2_SO_3_ (room temperature, RT, for 15 min), keeping the reaction mixture in agitation. Resulting oxidized GAC (GACox) was then purified and exchanged in water through tangential flow filtration (TFF) or PD-10 Desalting column (Cytiva Life Sciences, Marlborough, MA, USA; formerly GE Healthcare Life Sciences), as previously reported [16], and stored at 4 °C freeze-dried until conjugation. GAC (10 or 40 mg/mL) was conjugated to CRM_197_ (40 mg/mL) in the presence of 5 mg/mL of NaBH_3_CN at pH 8 in borate buffer. After 4 h or overnight (ON) mixing at 37 °C, the reaction mixture was diluted 1:10 with PBS, and unreacted aldehydic groups of GACox were quenched adding NaBH_4_ (NaBH_4_:GAC w/w ratio of 0.5 to 1), incubating for 2 h at 37 °C. Based on the reaction scale, conjugate was purified through TFF using a membrane with 50 kDa molecular weight cut-off, or by Amicon Ultra 30 kDa cut-off, as previously reported [16].

HPAEC-PAD was used to evaluate % of GlcNAc oxidized in GACox, by comparing GlcNAc to rhamnose (Rha) molar ratios before and after oxidation [34]. HPLC-SEC (TSKgel G3000 PWXL column) was used to check that no changes in GAC chain length had occurred after oxidation.

Purified conjugate was analyzed by micro-BCA, with bovine serum albumin (BSA) as a reference, following the manufacturer’s instructions (Thermo Scientific, Waltham, MA, USA), and HPAEC-PAD [34] for total protein and total GAC content, respectively, and to calculate the PS to protein ratio. GAC concentration was quantified by HPAEC-PAD based on Rha amount, as GlcNAc is impacted in the oxidation step. Free GAC was determined by HPAEC-PAD after conjugate co-precipitation with DOC [35]. The purified conjugate was analyzed by SDS-PAGE and HPLC-SEC analyses to verify conjugate formation, as previously described [16].

#### 2.2.2. GAC-GMMA Conjugate

Prior to oxidation and subsequent conjugation via reductive amination, *S. typhimurium* OAg-negative GMMA were concentrated at 10 mg/mL and exchanged in 100 mM acetate buffer pH 4.5. GMMA were oxidized with NaIO_4_ (15 mM in the final reaction mixture) for 30 min at 25 °C, and then, the reaction was quenched with Na_2_SO_3_ at a final concentration of 30 mM, mixing for 15 min at RT. Reductive amination, between the previously derivatized polysaccharide with a dihydrazide linker (i.e., ADH, [36]) and reactive aldehydes of oxidized sugar residues of GMMA lipopolysaccharide (LPS) core, was conducted ON at RT with GAC/GMMA/NaCNBH_3_ w/w/w ratio of 3:1:1. GAC-GMMA conjugate was then purified through centrifugal ultrafiltration using Amicon Ultra device with a membrane cut-off of 100 kDa (Merck, Darmstadt, Germany), and removal of unconjugated polysaccharide was verified via HPLC-SEC analysis with respect to a calibration curve of purified unconjugated polysaccharide (TSK gel G3000 PWXL column, refractive index detector). Total protein quantification in GAC-GMMA conjugate was estimated by micro-BCA analysis, while GAC amount estimation was based on Rha quantification through HPAEC-PAD analysis [37,38], as GlcNAc is also present in the LPS outer core of *S. typhimurium* OAg-negative GMMA. Conjugate size distribution was determined by DLS [39].

### 2.3. Formulation of Conjugates

Conjugates were diluted at the concentration of the study in saline and when needed formulated with Alhydrogel (0.4–2 mg/mL Al^3+^). Conjugate adsorption on Alhydrogel was evaluated by analyzing formulation supernatants by SDS-PAGE with silver staining detection following manufacturer’s instruction (SilverQuest Silver Staining kit, ThermoFisher Scientific, Waltham, MA, USA), after Alhydrogel removal by two sequential centrifugations (18,000 rcf, 15 min, 4 °C). It was verified that >90% of the conjugates was adsorbed on Alhydrogel.

### 2.4. Immunogenicity Studies in Mice and Rabbits

Mouse immunogenicity studies were performed in agreement with the European Directive 63/2010 on the protection of animals used for scientific purposes and its national transpositions, under the animal project 526/2020-PR, approved by the Italian Ministry of Health, in compliance with the legislation (Italian D.Lgs. 26/2014 and) at AAALAC-accredited GSK Animal Care Facility (Siena, Italy), and at NIBSC Animal Care Facility (South Mimms, UK), in compliance with the United Kingdom Animal (Scientific Procedures) ACT 1986.

In the first study, 5-week-old female CD1 mice (8 per group) were vaccinated intraperitoneally (IP) with 200 µL of GAC-GMMA and GAC-CRM_197_ conjugates at study day 0 and 28. Approximately 100 µL of blood samples (50 µL serum) was collected on day −1 (pooled sera) and on day 27 (individual sera), with final blood collection on day 42 (individual sera). In the second immunogenicity study in mice, 6–12-week-old female BalbC mice (8 per group) were injected subcutaneously (SC) with 200 µL of vaccine on study day 0 and 22. Approximately 100 µL blood samples (50 µL serum) were collected on day −1 (pooled sera) and on day 21 (individual sera) with final blood collection on day 36 (individual sera). The rabbit immunogenicity study was conducted in an AAALAC-accredited facility at Charles River Laboratories, in accordance with the European Directive 63/2010.

New Zealand White female rabbits (6 per group) were vaccinated intramuscularly (IM) with 500 µL of vaccine at study day 0, 21 and 35 [17]. Individual sera were collected on day 20 and day 34, with final blood collection on day 49.

### 2.5. ELISA for Anti-GAC IgG Response in Mice

Sera were assessed for anti-GAC specific IgG by enzyme-linked immunosorbent assay (ELISA) as previously described [16]. In brief, each mouse serum was run in triplicate at three different dilutions (1:100, 1:4000 and 1:160,000) in PBS containing 0.05% Tween 20 and 0.1% BSA. GAC-HSA was used as coating antigen at the concentration of 1 µg/mL in carbonate buffer pH 9.6. ELISA units were calculated based on an anti-antigen standard serum curve, with best fit determined by a five-parameter logistic equation. One ELISA unit was defined as the reciprocal of the standard serum dilution that gives an absorbance value equal to 1 in the assay. Individual mouse ELISA units and geometric mean of each group were reported in graphs.

For the NIBSC study, mouse sera were titrated in 11 doubling dilutions on the ELISA plate (coated with GAC-HSA), starting with 1/100 dilution, and antibody binding was detected using rabbit anti-mouse IgG-HRP antibodies (Sigma, Saint Louis, MO, USA). Each serum was tested in duplicate, and results were expressed as antibody titer = reciprocal of serum dilution giving OD of ≥0.5. Data are presented as scatter plot of individual mouse serum titer and GMT of each group.

To detect the level of IgG1 and IgG2a anti-GAC antibodies, ELISA was performed as above but using IgG subclass specific detection antibodies, e.g., goat anti-mouse IgG2a-HRP or goat anti-mouse IgG1-HRP (Abcam, Cambridge, UK). Results for IgG1 and IgG2a anti-GAC antibodies are reported as relative concentration (ng/mL), calculated by interpolating from standard curves generated from commercial mouse IgG1 and IgG2a myeloma proteins of known concentration (Sigma, Saint Louis, MO, USA) serially diluted, tested on plates coated with anti-IgG instead of GAC-HSA.

### 2.6. Luminex for Anti-GAC IgG Response in Rabbits

Anti-GAC-specific total IgG antibodies were determined using a multiplex bead-based methodology. Briefly, MagPlex-C magnetic carboxylated microspheres were conjugated to streptavidin and afterward coupled with purified biotinylated GAC. Individual rabbit sera were diluted eight times 3-fold apart, at starting dilution 1:100 in PBS pH 7.2 in 96-well round bottom white color plate. A standard curve made with polyclonal anti-GAC specific sera (eight points 3-fold serially diluted) was also run in duplicate in each 96-well plate. Antibody-coupled beads were mixed and added to the sera samples for 1 h at RT in 750 rpm shaking. Plate was afterward washed using a plate washer equipped with the magnetic holder on the plate carrier. Then, 50 µL/well of secondary antibody conjugated to phycoerythrin (PE) was added to the wells and incubated for 15 min at RT in 750 rpm shaking. Plate was afterward washed using a plate washer equipped with the magnetic holder on the plate carrier, and the beads were resuspended in 50 µL PBS. The resulting immune complexes were analyzed on the Luminex Bioplex-200 system. MFI values obtained for each point of each of the standard curves assayed were used to fit a 5PL curve by Bio-Plex Manager Software. An arbitrary titer of 100 Relative Luminex Units (RLU/mL) was assigned to the first dilution point of the standard curve for each antigen. The IgG RLU/mL of unknown test samples are derived by using their MFI value as input by comparison to the standard 5PL curve. The median of at least three dilution points within the acceptable range of the standard curve was calculate and represented the IgG RLU/mL of unknown test samples. For those samples for which only two or less dilutions fall within the acceptable range of the standard curve, the RLU was assigned as half of the lower limit of standard curve accuracy (calculated by the Bioplex software) multiplied by the lowest dilution of the sample tested.

### 2.7. Flow Cytometry (FACS)

For the GVGH study, GAS strain GAS51∆M1 was grown ON in Todd Hewitt broth plus Yeast extract at 37 °C and 5% CO_2_. Bacteria were pelleted at 4000× *g* for 5 min and washed with PBS. Bacteria were then blocked with PBS + 3% (*w*/*V*) BSA for 20 min and incubated with pooled mice or rabbit sera diluted 1:10,000 in PBS  +  1% (*w*/*V*) BSA for one hour.

For the NIBSC study, GAS strain NCTC 8198 was grown in Todd Hewitt broth plus Yeast extract at 37°C and 5% CO_2_ until reaching OD_600_ 0.4. Bacteria were centrifuged at 4000× *g* for 5 min, and the resulting pellet was washed with PBS. Bacteria were then blocked with PBS + 10% (*V*/*V*) goat serum for 20 min and incubated with pooled mouse sera diluted 1:4 in PBS + 10% (*V*/*V*) Goat serum and 0.1% (*w*/*V*) BSA at 4 °C for one hour.

After washes, samples were incubated with Alexa Fluor 647 anti-mouse IgG (Molecular Probes), Alexa Fluor 488 anti-rabbit IgG (Molecular Probes) or FITC anti-mouse IgG (Thermofisher Scientific, Waltham, MA, USA) diluted in PBS + 0.1% (*w*/*V*) BSA for 45 min at 4°C. Finally, bacteria were fixed with 4% (*w*/*V*) formaldehyde for 20 min.

Flow cytometry analyses were performed on a FACS Canto II flow cytometer (BD Biosciences). Results are reported as overlaid histograms with the relative fluorescence intensity on the X axis and the percentage of the maximum number of events on the Y axis.

### 2.8. Antibody Affinity Measurements

Affinity between purified GAC antigen and antibodies in GAC-CRM_197_ and GAC-GMMA rabbit sera was evaluated through the fully automated microfluidics-based platform Gyrolab [40]. The streptavidin-coated column was loaded with a 100 mg/mL biotinylated GAC solution in wash buffer (1 × PBS with 0.01% Tween 20) for use as capture antibody reagent. A working solution of 25 nM of Alexa Fluor 647-labeled detection antibodies was prepared in detection buffer (Rexxip F). The layout of capture antigens, detection antibodies, pooled rabbit sera (diluted in Rexxip A), as well as wash buffer in the plate was in accordance with the plate map automatically generated by the Gyrolab software after setting up the run conditions. All the plates were loaded into the instrument along with BioAffy 200 nL compact discs (CDs Gyros) and run using the fully automated microfluidics workstation with a 200–3W-001 Wizard method that performs three separate addition steps for capture, analyte and detection with spins and washes in between using PBS with 0.01% Tween 20 buffer Response units were calculated using the photomultiplier tube in the instrument at three percentages (1, 5 and 10%).

### 2.9. Techno-Economic Analysis

GMMA production, CRM_197_ production, the conjugation of GAC to GMMA, and the conjugation of GAC to CRM_197_ were modeled in SuperPro Designer Version 12, Build 3, from Intelligen, Inc. Supplementary Table S1 reports the input parameters and assumptions for techno-economic modeling in SuperPro Designer. The time between consecutive batches (cycle slack time) was set to 2 h in all production process models. Set-up time, clean in place and steam in place were scheduled in addition to the cycle slack time for each equipment. All production processes were modeled to operate 330 days per year. The number of campaigns per year was set to 1 in all the production process models. Production de-bottlenecking and process intensification scenarios were not modeled in this study for neither of the processes. The duration of a single batch was modeled at 88 h for GMMA production, 135 h for CRM_197_ production, 90 h for the GAC-GMMA conjugation process, and 70 h for the GAC-CRM_197_ conjugation process, based on the duration of each unit operation and procedure in the process. The annualized capital costs (CapEx), calculated using the straight-line depreciation method were included in the operating costs (OpEx). The labor cost for DS production processes (operated in batch mode) was calculated considering the detailed labor estimate, in function of the basic labor rate, benefits, operating supplies, supervision cost and administration cost. The GMMA titer was modeled at 0.2 g of GMMA per L of bioreactor working volume. The CRM_197_ titer was modeled at 2.5 g of CRM_197_ per L of bioreactor working volume [41]. A single production line was modeled per facility for all processes. The production of GAC was not modeled, and all results were expressed per unit amount of GAC. The losses in the downstream purification were modeled at 44% and 21.5% for CRM_197_ and GMMA, respectively. The GAC losses were modeled at 90% in the GAC-GMMA conjugation process and at 70% in the GAC-CRM_197_ conjugation process. The production costs and process performance for manufacturing these two conjugate vaccines were modeled at a production scale required to manufacture 500 million conjugate vaccine doses per year. For the GAC-GMMA conjugate vaccine production, it was assumed that 83 µg of GMMA and 249 µg of GAC are needed to obtain 25 µg of GAC-GMMA conjugate, a vaccine dose containing 25 µg of GAC-GMMA conjugate. For the GAC-CRM_197_ conjugate vaccine production, it was assumed that 83 µg of CRM_197_ and 83 µg of GAC are needed to obtain 25 µg of GAC-CRM_197_ conjugate, a vaccine dose containing 25 µg of GAC-CRM_197_ conjugate.

### 2.10. Statistical Analysis

Statistical analysis was performed using GraphPad Prism 7. To compare the immune response elicited by two different antigens, the Mann–Whitney two-tailed test was used, while the response induced by the same antigen after first injection vs. second or third injection was compared by Wilcoxon matched-pairs signed rank test.

## 3. Results

### 3.1. Synthesis and Characterization of GAC-CRM_197_ and GAC-GMMA Conjugates

**Figure 1:**
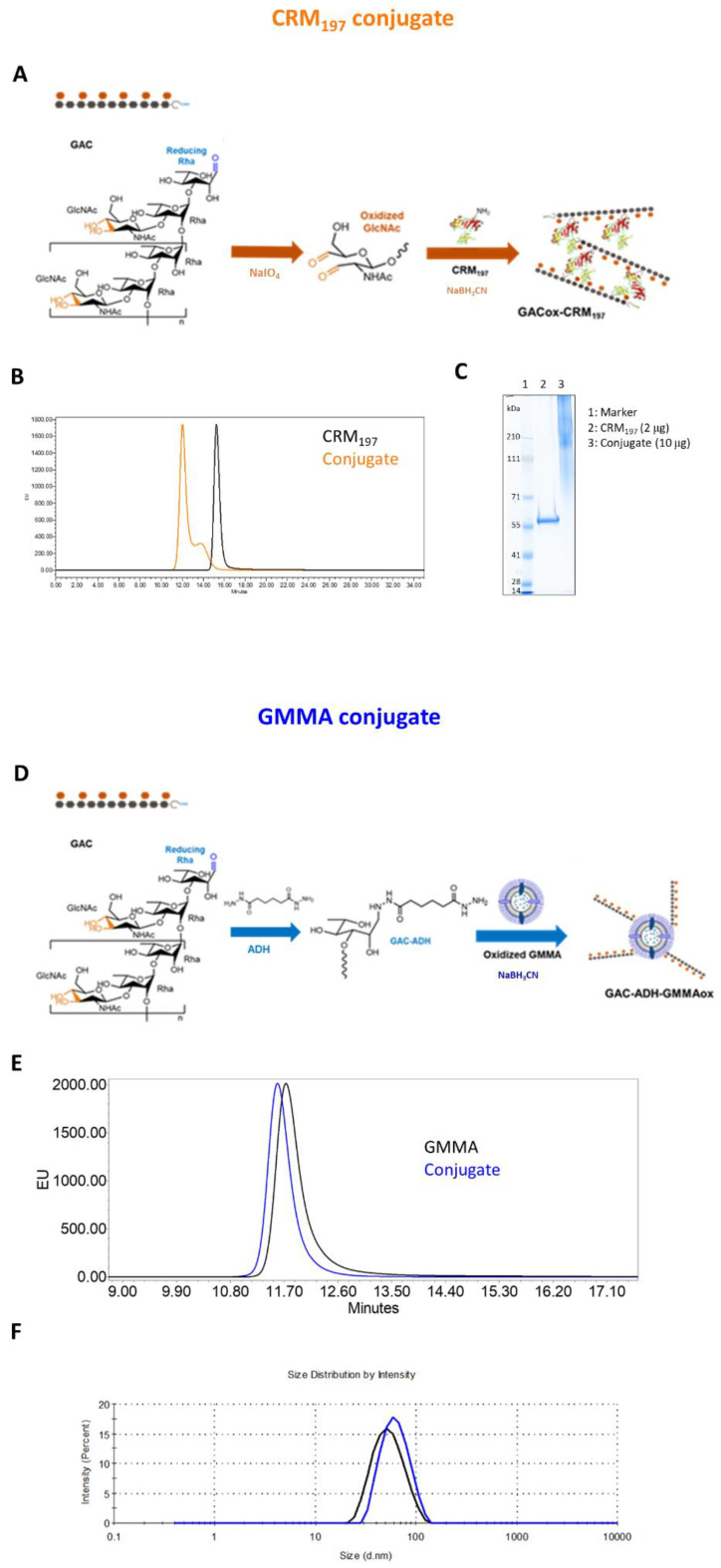
CRM_197_ and GMMA as carriers for GAC PS. (**A**) Reaction schemes: oxidized GAC was linked to lysines of CRM_197_ by reductive amination. (**D**) ADH-derivatized GAC was conjugated to oxidized LPS core sugars of GMMA via reductive amination chemistry. (**B**,**E**) Conjugates were characterized by HPLC-SEC analysis in comparison to the corresponding unconjugated carriers. (**C**) GAC-CRM_197_ and CRM_197_ were compared by SDS-PAGE, (**F**) with GAC-GMMA and GMMA by DLS.

GAC-CRM_197_ conjugate was obtained via reductive amination chemistry between the aldehyde groups randomly generated through sodium periodate oxidation along GAC chains and lysines of the carrier protein (Figure 1A, [16]). The oxidation that occurs at the vicinal diols of the GlcNAc side chain of GAC and by HPAEC-PAD analysis around 10% of oxidized GlcNAc was estimated. Conjugate formation was verified by HPLC-SEC and SDS-PAGE analyses, which showed a shift in the conjugate at higher molecular weight compared to the unconjugated protein (Figure 1B,C). GAC-CRM_197_ conjugates were characterized by a w/w ratio of 0.18–0.51 according to the reaction conditions used (in a previous study in mice, we had verified no impact of GAC to CRM_197_ ratio on immunogenicity in mice in this range). Analysis by DOC/HPAEC-PAD revealed <12% free saccharide in all lots of purified conjugates produced.

GAC was also conjugated to OAg-negative *S. typhimurium* GMMA, chosen as the alternative delivery system to be tested for this polysaccharide. In this case, lipooligosaccharide molecules on GMMA were oxidized (6%, 55% and 16% of oxidation for GlcNAc, Gal and Glc residues, respectively) and linked to GAC previously terminally derivatized with the ADH linker by reductive amination (Figure 1D). The GMMA conjugate resulted in a w/w ratio of polysaccharide to GMMA of 0.4 with a free saccharide content of less than 6.5%, estimated via HPLC-SEC analysis. Size distribution measured through DLS showed an increase of about 10 nm in GMMA particle hydrodynamic diameter after GAC conjugation, appreciable also by HPLC-SEC analysis from the lower retention time of the conjugate compared to that of the starting unconjugated GMMA (Figure 1E,F).

### 3.2. Immunogenicity in Mice and Rabbits of GAC-GMMA vs. GAC-CRM_197_ in the Presence of Alhydrogel

**Figure 2:**
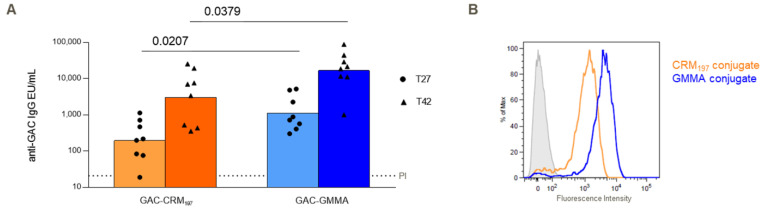
Comparative immunogenicity study of GAC-CRM_197_ and GAC-GMMA conjugates in mice. One experiment was performed: eight CD1 mice per group were immunized IP at days 0 and 28 with 1.5 μg GAC in the presence of 2 mg/mL of Alhydrogel (Al^3+^). Sera were collected at days −1 (PI, pre-immune), 27 and 42. (**A**) Summary graphs of anti-GAC IgG geometric mean units (bars) and individual antibody levels (dots) are reported. Mann–Whitney two-tailed test was used to compare the immune response elicited by the two conjugates that resulted as significantly different (*p* < 0.05). (**B**) FACS analysis against GAS strain GAS51∆M1 performed on Day 42 pooled sera. Grey-shaded histogram represents the pre-immune sera used as negative control.

GAC-GMMA and GAC-CRM_197_ conjugates, formulated with Alhydrogel, were tested in mice through IP immunization with the aim to compare their ability to induce anti-GAC-specific IgG response. Both 27 days after the first injection and 14 days after the second injection on day 28, GAC-GMMA elicited a significantly higher anti-GAC IgG response than GAC-CRM_197_ (*p* value = 0.0207 and 0.0379, respectively), as shown by the ELISA results in Figure 2A. For both conjugates, the response significantly increased after the second vaccination. FACS analysis of pooled sera from immunized mice against *S. pyogenes* showed stronger binding to the bacteria of anti-GAC antibodies induced by the GAC-GMMA conjugate with respect to GAC-CRM_197_ (Figure 2B).

**Figure 3:**
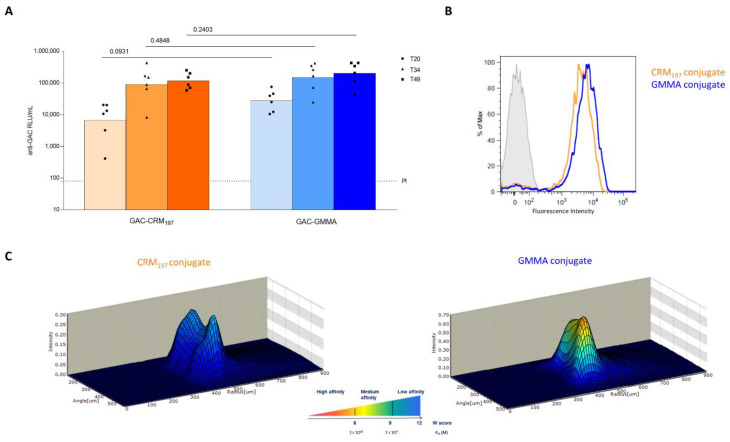
Comparative immunogenicity study of GAC-CRM_197_ and GAC-GMMA conjugates in rabbits. One experiment was performed: six New Zealand White female rabbits per group were immunized IM at days 0, 21 and 3 with 5 μg GAC in the presence of 0.7 mg/mL of Alhydrogel (Al^3+^). Sera were collected at days −1 (PI, pre-immune), 20, 34 and final bleed on day 49. (**A**) Summary graphs of anti-GAC IgG geometric mean units (bars) and individual antibody levels (dots) are reported. Mann–Whitney two-tailed test was used to compare the immune response elicited by the two conjugates that resulted as nonsignificantly different (*p* > 0.05). (**B**) FACS analysis GAS strain GAS51∆M1 performed on Day 49 pooled sera. Grey-shaded histogram represents the pre-immune sera used as negative control. (**C**) 3D view of antibody–antigen capture profiles (Gyrolab Viewer) showing the distribution of the antibodies in the column for the two conjugates.

GAC-GMMA and GAC-CRM_197_ conjugates were also compared in rabbits, using the same immunization scheme of previous studies with GAC-CRM_197_ in this animal model [17]. On day 49, two weeks after the third injection, no significant difference in anti-GAC IgG response was observed between the two conjugates (*p* value = 0.2403) and no differences were highlighted at earlier time points (*p* value = 0.0931 on day 20 and *p* value = 0.4848 on day 34, Figure 3A). In addition, FACS analysis did not reveal major differences between anti-GAC antibodies elicited by GAC-GMMA and by GAC-CRM_197_ conjugates for their ability to bind *S. pyogenes* bacteria (Figure 3B). However, antibody affinity measurements showed that antibodies from GAC-GMMA-immunized rabbits had higher affinity toward purified GAC antigen compared to those from GAC-CRM_197_, as shown by the higher 3D fluorescence intensity profiles for the GMMA conjugate, corresponding to lower K_D_ values (Figure 3C).

### 3.3. Immunogenicity Study in Mice of GAC-GMMA vs. GAC-CRM_197_ in Absence of Alhydrogel

**Figure 4:**
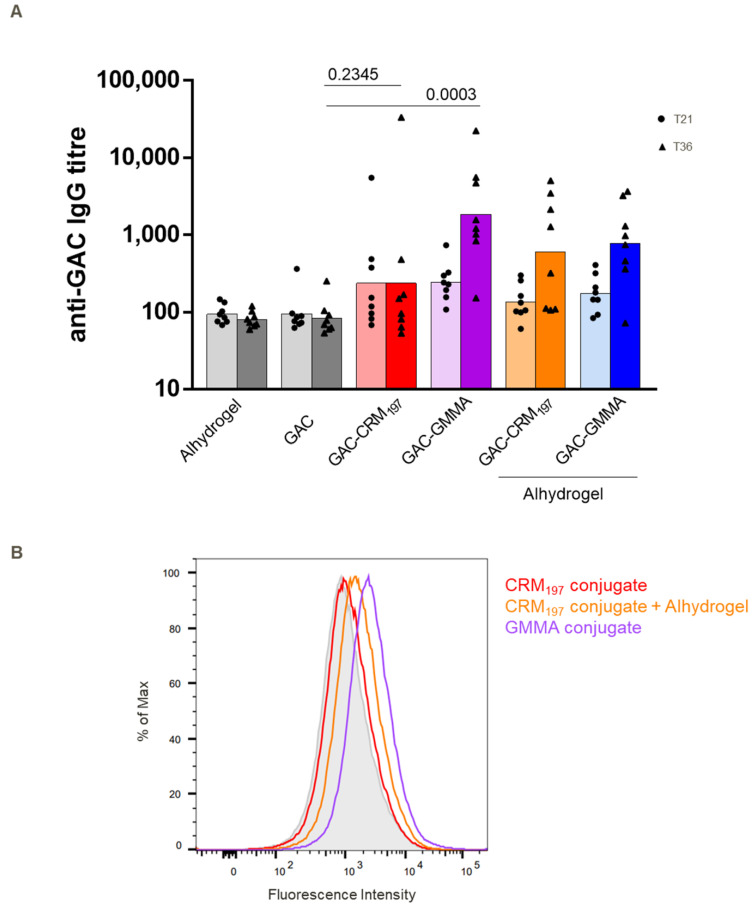
Comparative immunogenicity study of GAC-CRM_197_ and GAC-GMMA conjugates in mice with and without Alhydrogel. One experiment was performed: eight BalbC mice per group were immunized SC at days 0 and 22 with 2 μg GAC (with or without Alhydrogel, 0.4 mg/mL Al^3+^), and sera were collected on day 36. (**A**) Summary graph of anti-GAC IgG geometric mean titer (bars) and individual antibody levels (dots) are reported. Mann–Whitney two-tailed test was used to compare the immune response elicited by two different antigens, and *p* values for the comparison between unadjuvanted conjugates and GAC alone are highlighted (significant difference for *p* < 0.05). (**B**) FACS analysis against GAS strain NCTC 8198 performed on day 36 pooled sera. Grey-shaded histogram represents the sera from Alhydrogel group used as negative control.

To investigate GMMA intrinsic adjuvanticity, the two GAC conjugates were also compared in mice without Alhydrogel and to unconjugated GAC. As expected, unconjugated GAC gave a response no different from Alhydrogel (negative control). Two weeks after the second injection, the GAC-GMMA induced anti-GAC IgG response was significantly higher than unconjugated GAC (*p* = 0.0003), while GAC-CRM_197_ showed no difference in response (*p* = 0.2345), confirming the ability of GMMA to work as both a carrier and adjuvant (Figure 4). The presence of Alhydrogel increased the response elicited by GAC-CRM_197_, but not that elicited by GAC-GMMA. Sera from mice immunized with non-adjuvanted GAC-GMMA conjugate, with respect to those from GAC-CRM_197_ with or without Alhydrogel groups, produced a stronger fluorescence in flow cytometry using a *S. pyogenes* strain (Figure 4B), mirroring the data from the previous mice study (Figure 2B).

By looking at the IgG1 and IgG2a response induced by adjuvanted GAC-CRM_197_ and by GAC-GMMA without Alhydrogel, GAC-GMMA induced a significantly higher IgG2a response compared to the adjuvanted GAC-CRM_197_, with a corresponding higher IgG2a/IgG1 ratio (Table 1).

### 3.4. Techno-Economic Analysis: GMMA vs. CRM_197_ as Carrier

**Figure 5:**
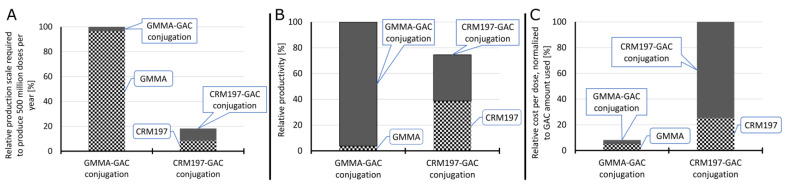
Techno-economic comparison of GMMA and CRM_197_ as a carrier for conjugate vaccine production. Relative scales of the GMMA production, CRM_197_ production, GAC-GMMA conjugation and GAC-CRM_197_ conjugation processes required to produce 500 million conjugate vaccine doses per year (**A**). The scale was expressed in fermenter working volume for GMMA and CRM_197_ production and in conjugation with reactor working volume for GAC-GMMA and GAC-CRM_197_ conjugation. In all cases, all unit operations were scaled proportionally to the fermenters and conjugation reactors. Relative productivity of the GMMA production, CRM_197_ production, GAC-GMMA conjugation and GAC-CRM_197_ conjugation processes (**B**). Productivity was expressed in doses produced per L of reactor scale per year. The relative drug substance production cost per dose, normalized to the amount of GAC used per dose of vaccine (**C**).

A process-modeling study was conducted to assess the techno-economic feasibility of GMMA as a carrier for conjugate vaccine production. For this, the conjugation of GAC to GMMA was compared with the conjugation of GAC to CRM_197_. The conjugation of GAC to GMMA is different from the conjugation of GAC to CRM_197_. In the former, GMMA is oxidized and GAC is activated with ADH linker, and then, the oxidized GMMA is conjugated to GAC-ADH (Figure 1D). In the latter, oxidized GAC is conjugated to CRM_197_ (Figure 1A). In addition, the production of CRM_197_ is different from GMMA production. Production process models have been built in SuperPro Designer to describe the large-scale production processes for performing the two different conjugation reactions (GAC-GMMA vs. GAC-CRM_197_) with the associated purification trains as well as for the large-scale production of both GMMA and CRM_197_. These production models compute the material and energy balances and the losses throughout the processes. Based on the material balances, the equipment requirements and their sizes were estimated. Next, the labor and utility requirements were also projected alongside the quality control costs. Scheduling of the production processes was also performed, which can provide valuable insights for process de-bottlenecking. From the equipment requirements, the facility requirements and the capital costs were also calculated. These techno-economic models capture all the capital and operating cost components associated with the production processes. This comparison assesses the differences in production rates, production throughputs, and production cost for manufacturing the GAC-GMMA drug substance in comparison to the GAC-CRM_197_ drug substance. These process-cost modeling results are expressed in relative percentages to each other (Figure 5). GAC-GMMA production requires the largest scales to produce vaccines for a high demand of 500 million doses per year. This is due to the high GMMA production scales required, which can be explained by the relatively low GMMA production yields of 0.2 g/L assumed in this study. If GMMA production yields are increased in the future, either by genetic engineering of the bacterial host or by process intensification, the scale requirements in case of GMMA production would decrease. Conversely, the productivity of GAC-GMMA production is higher than the productivity of GAC-CRM_197_ production. This productivity difference is driven by the high productivity of the GAC-GMMA conjugation process. The cost per dose of the GAC-GMMA production is over an order of magnitude lower than the GAC-CRM_197_ production cost per dose. The high GAC-CRM_197_ production costs are driven by the high costs in the conjugation process. However, the CRM_197_ production costs are also higher than the GMMA production costs, despite the higher CRM_197_ production yield and lower relative amount of CRM_197_ used in the conjugation process per vaccine dose.

## 4. Discussion

Currently, no licensed vaccine is available against GAS, which is estimated to cause globally about 500,000 annual deaths and 18.1 million cases, with the greatest burden due to RHD (at least 15.6 million) [3]. A subsequent review estimated between 1.96 and 2.21 million cases of RHD in Asian children 5 to 14 years of age [42]. Based on data collected from low- and middle-income countries, a study published in 2015 reported that RHD patients were young, mainly female, and with a high prevalence of major cardiovascular complications [43]. However, estimating the burden of GAS disease remains challenging, since the lack of high-quality data makes it difficult to confidently estimate the incidence and mortality from *S. pyogenes*. Better estimates of the disease will also facilitate further investment and development of a vaccine against GAS [44]. Thus far, only M protein-based candidate vaccines have been tested in clinical trials [45,46,47,48], but conserved protein antigens and surface polysaccharides have also been proposed as vaccine candidates in development [49]. Among them, GAC conjugate represents an attractive alternative vaccine candidate [45]. To date, a limited number of carrier proteins (mainly CRM_197_, DT and TT) have been used for licensed glycoconjugate vaccines, and there is increased concern for carrier-induced epitope suppression, which could result in reduced anti-carbohydrate immune response after patient-repeated exposure to a given carrier [19,50,51]. For this reason, different GAS protein antigens have been explored as possible carriers at the preclinical level [16]. There is also an increasing interest in investigating nanoparticle systems for polysaccharide presentation, due to the multivalent antigen presentation they offer and to their unique physico-chemical properties [19].

In this work, GMMA have been evaluated as alternative carriers for GAC with respect to the well-known non-toxic mutant of diphtheria toxin, CRM_197_ [25]. In previous studies, we have shown the ability of GMMA to elicit similar or enhanced anti-saccharide immune responses in respect to more traditional glycoconjugates with the CRM_197_ carrier protein, by using *Salmonella* O-antigens, meningococcal oligosaccharides and *Shigella flexneri* 6 O-antigen as model polysaccharides [28,52].

The two GAC conjugates were both produced through reductive amination chemistry: in the case of GAC-CRM_197_, GAC was randomly oxidized prior to conjugation with CRM_197_, while for the GMMA conjugate, GAC was terminally derivatized with ADH and then linked to oxidized sugars on the GMMA surface. From a techno-economic analysis, the GAC-GMMA conjugation process was more productive with respect to that of GAC-CRM_197_, leading to a cost difference per dose of more than one order of magnitude between the two candidate vaccines. Even if CRM_197_ is produced with a higher yield and is used in a lower relative amount in the conjugation process per vaccine dose, the production cost of GMMA is really unbeatable, making the GMMA conjugate more attractive for LMIC.

Overall, the results in mice and rabbits confirm the ability of GMMA to work well as a carrier for GAC, with the ability to induce anti-GAC IgG responses similar to CRM_197_. GMMA contain numerous pathogen-associated molecular patterns that, upon interaction with pattern recognition receptors expressed on mammalian cells, rapidly trigger pro-inflammatory cytokine and chemokine cascades, which may be the basis of GMMA self-adjuvanticity [53]. When testing in mice the two conjugates in absence of Alhydrogel, only the GMMA conjugate induced a response significantly higher than unconjugated GAC, differently from GAC-CRM_197_ (Figure 4). In addition, GAC-GMMA induced significantly higher anti-GAC IgG2a compared to the adjuvanted GAC-CRM_197_ (Table 1). Thus, the nature of the response elicited by the two conjugates was different: while GAC-CRM_197_ mainly stimulated a Th2 response (associated with the induction of IgG1), GMMA engaged both Th2 and Th1 (associated with IgG2a production) immunity. Adjuvants can induce changes in the Th1-Th2 balance, and according to the literature, aluminum hydroxide has been shown to drive the immune response toward the Th2 pattern [54,55,56]. In line with our results, a conjugate vaccine of meningococcal group C polysaccharides with a particulate carrier protein such as HBc VLPs (full-length hepatitis B core antigen virus-like particles) promoted a shift to a Th1 cellular immune-type response, as shown by the increased production of the IgG2a subclass [57]. The particulate nature confers to HBc VLP’s inherent adjuvant properties that enhance cell-mediated immune response [58].

In addition, by flow cytometry, GAC-GMMA sera demonstrated greater binding capacity toward different *S. pyogenes* strains.

Recently, it has been also shown that GMMA, when compared with soluble proteins, are more efficiently taken up in vivo by B cells, and this preferential targeting may increase antigen-processing efficiency, thereby enhancing immune response [59,60].

In conclusion, GMMA, due to their intrinsic adjuvant properties, could work both as a carrier and adjuvant for GAC PS. We have shown that GMMA are able to increase GAC response in the absence of Alhydrogel, provide a switch of IgG subclasses with increased affinity and lower costs compared to a traditional carrier protein such as CRM_197_. Additional studies will need to be performed to verify the opsonizing activity of anti-GAC-GMMA antibodies and compare GAC-GMMA with other vaccines in development against GAS by use of a challenge model. Furthermore, recent publications have shown that OMV are able to promote a mucosal immune response [61,62]. We also aim to verify this for GAC-GMMA, performing additional studies to verify the ability of GAC-GMMA to elicit mucosal immunity, always in comparison to GAC-CRM_197_. If the results will be confirmed, the use of GMMA as a carrier could constitute an additional advantage for a vaccine targeting a mucosal pathogen such as GAS. Oral immunization could provide greater protection and would also have the added benefits of being cheap and easy to perform, properties that could be particularly attractive for a vaccine targeting low- and middle-income countries.

## Figures and Tables

**Table 1:** IgG1 and IgG2 in mice sera generated by adjuvanted GAC-CRM197 and GAC GMMA without Alhydrogel. Results are presented as group average concentration (ng/mL) + SD and average ratio of IgG2a:IgG1. Statistical significance between the groups was assessed by Mann–Whitney test with *p* < 0.05 considered significant.

Group	Average Relative Concentration in ng/mL + SD		IgG2a:IgG1 Ratio
	IgG1	IgG2a	
**GAC-CRM_197_ Alhydrogel**	3017 ± 2670	20 ± 36	0.0083
**GAC-GMMA**	3049 ± 4941	1101 ± 1352	1.3886
**Significance** **between** **groups**	*p* > 0.05	*p* < 0.01	*p* < 0.01

## Data Availability

The authors declare that data are contained within the article and in the supplementary material.

## Supplementary Materials

The following supporting information can be downloaded at: https://www.mdpi.com/article/10.3390/vaccines10071034/s1, Table S1: Input parameters and assumptions used for techno-economic modeling in SuperPro Designer.

## References

[1] Ralph A.P., Carapetis J.R., Chhatwal G.S. (2013). Group A Streptococcal Diseases and Their Global Burden. Host-Pathogen Interactions in Streptococcal Diseases.

[2] Mitchell T.J. (2003). The pathogenesis of streptococcal infections: From Tooth decay to meningitis. Nat. Rev. Microbiol..

[3] Carapetis J.R., Steer A.C., Mulholland E.K., Weber M. (2005). The global burden of group A streptococcal diseases. Lancet Infect. Dis..

[4] Marijon E., Mirabel M., Celermajer D.S., Jouven X. (2012). Rheumatic heart disease. Lancet.

[5] Watkins D.A., Johnson C.O., Colquhoun S.M., Karthikeyan G., Beaton A., Bukhman G., Forouzanfar M.H., Longenecker C.T., Mayosi B.M., Mensah G.A. (2017). Global, Regional, and National Burden of Rheumatic Heart Disease, 1990–2015. N. Engl. J. Med..

[6] Dooling K.L., Shapiro D.J., Van Beneden C., Hersh A.L., Hicks L.A. (2014). Overprescribing and Inappropriate Antibiotic Selection for Children With Pharyngitis in the United States, 1997–2010. JAMA Pediatr..

[7] Micoli F., Bagnoli F., Rappuoli R., Serruto D. (2021). The role of vaccines in combatting antimicrobial resistance. Nat. Rev. Microbiol..

[8] Sanderson-Smith M., De Oliveira D.M.P., Guglielmini J., McMillan D.J., Vu T., Holien J.K., Henningham A., Steer A.C., Bessen D.E., Dale J.B. (2014). A Systematic and Functional Classification of *Streptococcus pyogenes* That Serves as a New Tool for Molecular Typing and Vaccine Development. J. Infect. Dis..

[9] Steer A.C., Law I., Matatolu L., Beall B.W., Carapetis J.R. (2009). Global emm type distribution of group A streptococci: Systematic review and implications for vaccine development. Lancet Infect. Dis..

[10] McCarty M. (1952). The lysis of group A hemolytic streptococci by extracellular enzymes of Streptomyces albus. II. Nature of the cellular substrate attacked by the lytic enzymes. J. Exp. Med..

[11] Cunningham M.W. (2000). Pathogenesis of Group A Streptococcal Infections. Clin. Microbiol. Rev..

[12] Van Sorge N.M., Cole J.N., Kuipers K., Henningham A., Aziz R.K., Kasirer-Friede A., Lin L., Berends E.T.M., Davies M.R., Dougan G. (2014). The Classical Lancefield Antigen of Group A *Streptococcus* Is a Virulence Determinant with Implications for Vaccine Design. Cell Host Microbe.

[13] Sabharwal H., Michon F., Nelson D., Dong W., Fuchs K., Manjarrez R.C., Sarkar A., Uitz C., Viteri-Jackson A., Suarez R.S.R. (2006). Group A *Streptococcus* (GAS) Carbohydrate as an Immunogen for Protection against GAS Infection. J. Infect. Dis..

[14] Salvadori L.G., Blake M.S., McCarty M., Tai J.Y., Zabriskie J.B. (1995). Group A *Streptococcus*-Liposome Elisa Antibody Titers To Group A Polysaccharide And Opsonophagocytic Capabilities Of The Antibodies. J. Infect. Dis..

[15] Rappuoli R. (2018). Glycoconjugate vaccines: Principles and mechanisms. Sci. Transl. Med..

[16] Di Benedetto R., Mancini F., Carducci M., Gasperini G., Moriel D.G., Saul A., Necchi F., Rappuoli R., Micoli F. (2020). Rational Design of a Glycoconjugate Vaccine against Group A *Streptococcus*. Int. J. Mol. Sci..

[17] Kabanova A., Margarit I., Berti F., Romano M.R., Grandi G., Bensi G., Chiarot E., Proietti D., Swennen E., Cappelletti E. (2010). Evaluation of a Group A *Streptococcus* synthetic oligosaccharide as vaccine candidate. Vaccine.

[18] McCluskie M.J., Evans D.M., Zhang N., Benoit M., McElhiney S.P., Unnithan M., DeMarco S.C., Clay B., Huber C., Deora A. (2016). The effect of preexisting anti-carrier immunity on subsequent responses to CRM197 or Qb-VLP conjugate vaccines. Immunopharmacol. Immunotoxicol..

[19] Micoli F., Costantino P., Adamo R. (2018). Potential targets for next generation antimicrobial glycoconjugate vaccines. FEMS Microbiol. Rev..

[20] Bensi G., Mora M., Tuscano G., Biagini M., Chiarot E., Bombaci M., Capo S., Falugi F., Manetti A.G.O., Donato P. (2012). Multi High-Throughput Approach for Highly Selective Identification of Vaccine Candidates: The Group A *Streptococcus* Case. Mol. Cell. Proteom..

[21] Wang G., Zhao J., Zhao Y., Wang S., Feng S., Gu G. (2021). Immunogenicity Assessment of Different Segments and Domains of Group A Streptococcal C5a Peptidase and Their Application Potential as Carrier Protein for Glycoconjugate Vaccine Development. Vaccines.

[22] Furuyama N., Sircili M.P. (2021). Outer Membrane Vesicles (OMVs) Produced by Gram-Negative Bacteria: Structure, Functions, Biogenesis, and Vaccine Application. BioMed. Res. Int..

[23] Tan K., Li R., Huang X., Liu Q. (2018). Outer Membrane Vesicles: Current Status and Future Direction of These Novel Vaccine Adjuvants. Front. Microbiol..

[24] van der Pol L., Stork M., van der Ley P. (2015). Outer membrane vesicles as platform vaccine technology. Biotechnol. J..

[25] Mancini F., Micoli F., Necchi F., Pizza M., Berlanda Scorza F., Rossi O. (2021). GMMA-Based Vaccines: The Known and The Unknown. Front. Immunol..

[26] Kis Z., Shattock R., Shah N., Kontoravdi C. (2019). Emerging Technologies for Low-Cost, Rapid Vaccine Manufacture. Biotechnol. J..

[27] Micoli F., Rondini S., Alfini R., Lanzilao L., Necchi F., Negrea A., Rossi O., Brandt C., Clare S., Mastroeni P. (2018). Comparative immunogenicity and efficacy of equivalent outer membrane vesicle and glycoconjugate vaccines against nontyphoidal *Salmonella*. Proc. Natl. Acad. Sci. USA.

[28] Micoli F., Alfini R., Di Benedetto R., Necchi F., Schiavo F., Mancini F., Carducci M., Palmieri E., Balocchi C., Gasperini G. (2020). GMMA Is a Versatile Platform to Design Effective Multivalent Combination Vaccines. Vaccines.

[29] Micoli F., Adamo R., Costantino P. (2018). Protein Carriers for Glycoconjugate Vaccines: History, Selection Criteria, Characterization and New Trends. Molecules.

[30] Wang S., Zhao Y., Wang G., Feng S., Guo Z., Gu G. (2020). Group A *Streptococcus* Cell Wall Oligosaccharide-Streptococcal C5a Peptidase Conjugates as Effective Antibacterial Vaccines. ACS Infect. Dis..

[31] Pancholi V., Fischetti V.A. (1988). Isolation and characterization of the cell-associated region of group A streptococcal M6 protein. J. Bacteriol..

[32] Datsenko K.A., Wanner B.L. (2000). One-step inactivation of chromosomal genes in *Escherichia coli* K-12 using PCR products. Proc. Natl. Acad. Sci. USA.

[33] Micoli F., Alfini R., Giannelli C., Bidmos F., Bossé J., Langford P. (2022). Methods for Assessment of OMV/GMMA Quality and Stability. Bacterial Vaccines: Methods and Protocols.

[34] Pitirollo O., Micoli F., Necchi F., Mancini F., Carducci M., Adamo R., Evangelisti C., Morelli L., Polito L., Lay L. (2020). Gold nanoparticles morphology does not affect the multivalent presentation and antibody recognition of Group A *Streptococcus* synthetic oligorhamnans. Bioorganic Chem..

[35] Lei Q.P., Lamb D.H., Heller R., Pietrobon P. (2000). Quantitation of low level unconjugated polysaccharide in tetanus toxoid-conjugate vaccine by HPAEC/PAD following rapid separation by deoxycholate/HCl. J. Pharm. Biomed. Anal..

[36] Micoli F., Giannelli C., Di Benedetto R., Pfeifer B.A., Hill A. (2021). O-Antigen Extraction, Purification, and Chemical Conjugation to a Carrier Protein. Vaccine Delivery Technology: Methods and Protocols.

[37] De Benedetto G., Alfini R., Cescutti P., Caboni M., Lanzilao L., Necchi F., Saul A., MacLennan C.A., Rondini S., Micoli F. (2017). Characterization of O-antigen delivered by Generalized Modules for Membrane Antigens (GMMA) vaccine candidates against nontyphoidal Salmonella. Vaccine.

[38] Micoli F., Ravenscroft N., Cescutti P., Stefanetti G., Londero S., Rondini S., MacLennan C.A. (2014). Structural analysis of O-polysaccharide chains extracted from different Salmonella Typhimurium strains. Carbohydr. Res..

[39] De Benedetto G., Cescutti P., Giannelli C., Rizzo R., Micoli F. (2017). Multiple Techniques for Size Determination of Generalized Modules for Membrane Antigens from *Salmonella typhimurium* and *Salmonella enteritidis*. ACS Omega.

[40] Myzithras M., Bigwarfe T., Waltz E., Li H., Ahlberg J., Rybina I., Low S., Kenny C.H., Miglietta J., Kroe-Barrett R. (2018). Optimizing NBE PK/PD assays using the Gyrolab Affinity Software; conveniently within the bioanalyst’s existing workflow. Bioanalysis.

[41] Goffin P., Dewerchin M., De Rop P., Blais N., Dehottay P. (2017). High-yield production of recombinant CRM197, a non-toxic mutant of diphtheria toxin, in the periplasm of Escherichia coli. Biotechnol. J..

[42] Carapetis J.R. (2008). Rheumatic Heart Disease in Asia. Circulation.

[43] Zühlke L., Engel M.E., Karthikeyan G., Rangarajan S., Mackie P., Cupido B., Mauff K., Islam S., Joachim A., Daniels R. (2015). Characteristics, complications, and gaps in evidence-based interventions in rheumatic heart disease: The Global Rheumatic Heart Disease Registry (the REMEDY study). Eur. Heart J..

[44] Sims Sanyahumbi A., Colquhoun S., Wyber R., Carapetis J.R., Ferretti J.J., Stevens D.L., Fischetti V.A. (2016). Global Disease Burden of Group A *Streptococcus*. Streptococcus pyogenes: Basic Biology to Clinical Manifestations.

[45] Vekemans J., Gouvea-Reis F., Kim J.H., Excler J.-L., Smeesters P.R., O’Brien K.L., Van Beneden C.A., Steer A.C., Carapetis J.R., Kaslow D.C. (2019). The Path to Group A *Streptococcus* Vaccines: World Health Organization Research and Development Technology Roadmap and Preferred Product Characteristics. Clin. Infect. Dis..

[46] Dale J.B., Penfound T.A., Chiang E.Y., Walton W.J. (2011). New 30-valent M protein-based vaccine evokes cross-opsonic antibodies against non-vaccine serotypes of group A streptococci. Vaccine.

[47] Postol E., Alencar R., Higa F.T., Freschi de Barros S., Demarchi L.M.F., Kalil J., Guilherme L. (2013). StreptInCor: A Candidate Vaccine Epitope against S. pyogenes Infections Induces Protection in Outbred Mice. PLoS ONE.

[48] Sekuloski S., Batzloff M.R., Griffin P., Parsonage W., Elliott S., Hartas J., O’Rourke P., Marquart L., Pandey M., Rubin F.A. (2018). Evaluation of safety and immunogenicity of a group A *Streptococcus* vaccine candidate (MJ8VAX) in a randomized clinical trial. PLoS ONE.

[49] Steer A.C., Carapetis J.R., Dale J.B., Fraser J.D., Good M.F., Guilherme L., Moreland N.J., Mulholland E.K., Schodel F., Smeesters P.R. (2016). Status of research and development of vaccines for *Streptococcus pyogenes*. Vaccine.

[50] Avci F., Berti F., Dull P., Hennessey J., Pavliak V., Prasad A.K., Vann W., Wacker M., Marcq O., Papasianm C.J. (2019). Glycoconjugates: What It Would Take To Master These Well-Known yet Little-Understood Immunogens for Vaccine Development. mSphere.

[51] Dagan R., Poolman J., Siegrist C.-A. (2010). Glycoconjugate vaccines and immune interference: A review. Vaccine.

[52] Micoli F., Alfini R., Di Benedetto R., Necchi F., Schiavo F., Mancini F., Carducci M., Oldrini D., Pitirollo O., Gasperini G. (2021). Generalized Modules for Membrane Antigens as Carrier for Polysaccharides: Impact of Sugar Length, Density, and Attachment Site on the Immune Response Elicited in Animal Models. Front. Immunol..

[53] Mancini F., Rossi O., Necchi F., Micoli F. (2020). OMV Vaccines and the Role of TLR Agonists in Immune Response. Int. J. Mol. Sci..

[54] McKee A.S., Munks M.W., Marrack P. (2007). How Do Adjuvants Work? Important Considerations for New Generation Adjuvants. Immunity.

[55] Marrack P., McKee A.S., Munks M.W. (2009). Towards an understanding of the adjuvant action of aluminium. Nat. Rev. Immunol..

[56] HogenEsch H. (2013). Mechanism of Immunopotentiation and Safety of Aluminum Adjuvants. Front. Immunol..

[57] Xu L., Li Z., Su Z., Yang Y., Ma G., Yu R., Zhang S. (2019). Development of meningococcal polysaccharide conjugate vaccine that can elicit long-lasting and strong cellular immune response with hepatitis B core antigen virus-like particles as a novel carrier protein. Vaccine.

[58] Roose K., Baets S.D., Schepens B., Saelens X. (2013). Hepatitis B core–based virus–like particles to present heterologous epitopes. Expert Rev. Vaccines.

[59] Suan D., Sundling C., Brink R. (2017). Plasma cell and memory B cell differentiation from the germinal center. Curr. Opin. Immunol..

[60] Yuseff M.-I., Pierobon P., Reversat A., Lennon-Duménil A.-M. (2013). How B cells capture, process and present antigens: A crucial role for cell polarity. Nat. Rev. Immunol..

[61] Micoli F., MacLennan C.A. (2020). Outer membrane vesicle vaccines. Semin. Immunol..

[62] van der Ley P.A., Zariri A., van Riet E., Oosterhoff D., Kruiswijk C.P. (2021). An Intranasal OMV-Based Vaccine Induces High Mucosal and Systemic Protecting Immunity Against a SARS-CoV-2 Infection. Front. Immunol..

