# Supplementary material for: GMMA as an Alternative Carrier for a Glycoconjugate Vaccine against Group A Streptococcus
Source: Vaccines (Basel). 2022 Jun 28;10(7):1034. doi: 10.3390/vaccines10071034 (PMC9324507; doi:10.3390/vaccines10071034)
Supplement: Supplementary file 1 [file vaccines-10-01034-s001.zip › vaccines-1724334-supplementary.pdf]

Supplementary material

GMMA as an Alternative Carrier for a Glycoconjugate Vaccine against Group A *Streptococcus*

Elena Palmieri [1], Zoltán Kis [2,3], James Ozanne [4], Roberta Di Benedetto [1], Beatrice Ricchetti [1], Luisa Massai [1], Martina Carducci [1], Davide Oldrini [1], Gianmarco Gasperini [1], Maria Grazia Aruta [1], Omar Rossi [1], Cleo Kontoravdi [2], Nilay Shah [2], Fatme Mawas [4], Francesca Micoli [1,*]

[1] GSK Vaccines Institute for Global Health (GVGH), Via Fiorentina 1, 53100 Siena, Italy; elena.x.palmieri@gsk.com (E.P.); roberta.x.di-benedetto@gsk.com (R.D.B.); beatrice.x.ricchetti@gsk.com (B.R.); luisa.x.massai@gsk.com (L.M.); martina.x.carducci@gsk.com (M.C.); davide.x.oldrini@gsk.com (D.O.); gianmarco.x.gasperini@gsk.com (G.G.); maria-grazia.x.aruta@gsk.com (M.G.A.); omar.x.rossi@gsk.com (O.R.)

[2] The Sargent Centre for Process Systems Engineering, Department of Chemical Engineering, Imperial College London, South Kensington Campus, London SW7 2AZ, UK; cleo.kontoravdi98@imperial.ac.uk (C.K.); n.shah@imperial.ac.uk (N.S.)

[3] Department of Chemical and Biological Engineering, The University of Sheffield, Mappin Street, Sheffield S1 3JD, UK; z.kis@sheffield.ac.uk (Z.K.)

[4] The National Institute for Biological Standards and Control (NIBSC), South Mimms EN6 3QG, UK; James.Ozanne@nibsc.org (J.O.); Fatme.Mawas@nibsc.org (F.M.)

* Correspondence: francesca.x.micoli@gsk.com; Tel.: +39-0577-539087

Supplementary Table S1. Input parameters and assumptions used for techno-economic modelling in SuperPro Designer.

Parameter use	Parameter class	Parameter name	Value	Unit
CapEx calculation	Direct Cost (DC)	Piping Cost	35	% of TEPC
		Instrumentation Cost	40	% of TEPC
		Insulation Cost	03	% of TEPC
		Electrical Facilities Cost	10	% of TEPC
		Buildings Cost	250	% of TEPC
		Yard Improvement Cost	15	% of TEPC
		Auxiliary Facilities Cost	40	% of TEPC
		Unlisted Equipment Purchase Cost (UEPC)	30	% of TEPC
		Unlisted Equipment Installation Cost	50	% of UEPC
	Indirect Cost (IC)	Engineering Cost	25	% of DC
		Construction Cost	35	% of DC
	Other Cost (OC)	Contractor's Fee	5	% of (IC + DC)
		Contingency	10	% of (IC + DC)
	Miscellaneous	Working Capital – to cover expenses for	30	days
		Start-up and Validation Costs	30	% of DFC
		Up front R&D	0	US$
		Up front royalties	0	US$
		Maintenance: equipment specific multipliers		

OpEx calculation	Facility dependent	Depreciation: contribution from each equipment's undepreciated purchase cost		
		Insurance	1	% of DFC
		Local taxes	2	% of DFC
		Factory expenses	5	% of DFC
	Labour	Basic operator labour rate (BOLR)	22	USD × hour^{-1}
		Benefits factor	40	% of BOLR
		Operating supplies factor	10	% of BOLR
		Supervision factor	20	% of BOLR
		Administration factor	60	% of BOLR
		Lumped operator labour rate	75	USD × hour^{-1}
		Adjusted basic operator labour rate*	50.6	USD × hour^{-1}
		Direct labour time utilisation - batch	60	%
		Direct labour time utilisation - continuous	70	%
	Lab, QC, QA	Laboratory, quality control, quality assurance	15	% TLC
	Utilities	Standard electricity	0.1	US$ × (kW×h)$^{-1}$
		Chilled water	0.4	US$ × tonne^{-1}
		Cooled water	0.1	US$ × tonne^{-1}
		Steam	12	US$ × tonne^{-1}
	Miscellaneous	Fixed R&D	0	US$ × year^{-1}
		Variable R&D	0	US$ × g MP^{-1}
		On-going process validation	0	US$ × year^{-1}
		Other fixed	0	US$ × year^{-1}
		Other variable	0	US$ × g MP^{-1}
Overall economic evaluation	Time valuation	Construction period	20	months
		Start-up period	4	months
		Project lifetime	20	years
		Inflation	4	%
		NPV interest - Low	7	%
		NPV interest - Medium	9	%
		NPV interest - High	11	%
	Financing	Loan interest for DFC	9	%
		Loan interest for working capital	12	%
		Loan interest for up front R&D	12	%
		Loan interest for up front royalties	12	%
		Loan period for DFC	10	years
		Loan period for working capital	6	years
		Loan period for up front R&D	6	years
		Loan period for up front royalties	6	years
		DFC outlay for 1st year	30	% of DFC
		DFC outlay for 2nd year	40	% of DFC
		DFC outlay for 3rd year	30	% of DFC
		DFC outlay for 4th year	0	% of DFC
		DFC outlay for 5th year	0	% of DFC
		Straight line depreciation period	10	years
		Salvage value	5	% of DFC
	Production level	Operating capacity for each year	100	%
		Product failure rate	5	%

		Disposal cost	0	US$ × g MP^{-1}
	Miscellaneous	Income tax	40	%
		Fixed advertising and selling expenses	0	US$ × $year^{-1}$
		Variable advertising and selling expenses	0	US$ × g MP^{-1}
		Variable running royalty expenses	0	US$ × g MP^{-1}

Abbreviations used in Supplementary Table 3: CapEx – capital expenditure; OpEx – operating expense; TEPC – total equipment purchase cost; UEPC – unlisted equipment purchase cost; DFC – direct fixed capital; DC – direct cost; IC – indirect cost; OC – other cost; TLC – total labour costs; BOLR – basic operator labour rate; g MP – gram of main product.

*calculated based on benefits, operating supplies, supervision cost and administration cost.
